# Anaphylaxis to topical *Aloe vera* in a birch pollen allergic child

**DOI:** 10.1186/2045-7022-4-S2-P45

**Published:** 2014-03-17

**Authors:** Eva-Maria Varga, Merima Bublin, Ernst Eber, Heimo Breiteneder

**Affiliations:** 1 Medical University Graz, Dep. of Pediatrics, Graz, Austria; 2 Medical University Vienna, Dep. of Pathophysiology and Allergy Research, Vienna, Austria

## Background

Ingredients of aloe vera leaves are being increasingly used for their anti-inflammatory and slimming properties. However, serious allergic reactions are very rare and only occasionally reported. Here, we present an eleven year old girl with anaphylaxis within minutes after topical administration of aloe vera to a skin lesion. On admission, the girl showed no signs of respiratory or cardiovascular compromise after having been stabilized by her GP. Apart from mild allergic rhinitis symptoms during the birch pollen season, she had no other atopic disease. The family history revealed respiratory allergies in the mother and anaphylaxis to insect stings in the father.

## Methods and results

Allergy tests showed elevated total IgE levels (67.8 kU/l) in the ImmunoCAP® as well as allergen-specific IgE to rPhlp 1, nPhl p 4 and rPhl p 5, nAmb a 1 , rBet v 1 and cross-reactive sensitization to nuts and to the CCD marker nMUXF3 in the ISAC®. Sera from our patient and from seven additional allergic individuals were tested by ELISA for IgE-binding to aloe vera protein extract, Bet v 1 and two glycoproteins, HRP and Api g 5 (HRP-horse radish peroxidase and Api g 5 in celery). Six out of seven control sera were sensitized to Bet v 1 and one was allergic to peanut and sensitized to cross-reactive carbohydrate determinants (CCDs). Additionally, aloe vera protein-induced mediator release was tested in all patients using a rat basophil leukemia cell(RBL) assay. Whereas all birch pollen allergics showed strong IgE-binding to Bet v 1, IgE- binding to aloe vera was only observed in our patient and in two other serum samples, one from a birch pollen- and one from a peanut-allergic patient. Interestingly, all three patients showed much stronger binding to Api g 5 and HRP, as compared to the aloe vera extract. RBL assay showed a strong and dose-dependent mediator release of 60% in our patient and of 40% and 20% in the two patients with IgE positivity to Aloe vera.

## Conclusion

In summary, we believe that IgE antibodies to CCDs or Bet v 1 homologues in aloe vera may induce serious allergic reactions in atopic individuals.

